# Development of targeted therapy therapeutics to sensitize triple-negative breast cancer chemosensitivity utilizing bacteriophage phi29 derived packaging RNA

**DOI:** 10.1186/s12951-020-00758-4

**Published:** 2021-01-07

**Authors:** Long Zhang, Chaofeng Mu, Tinghong Zhang, Dejun Yang, Chenou Wang, Qiong Chen, Lin Tang, Luhui Fan, Cong Liu, Jianliang Shen, Huaqiong Li

**Affiliations:** 1 School of Biomedical Engineering, School of Ophthalmology & Optometry and Eye Hospital, Wenzhou Medical University, Wenzhou, 325035 Zhejiang People’s Republic of China; 2 Engineering Research Center of Clinical Functional Materials and Diagnosis & Treatment Devices of Zhejiang Province, Wenzhou Institute, University of Chinese Academy of Sciences, Wenzhou, 325011 Zhejiang People’s Republic of China; 3 Department of Pharmaceutics, College of Pharmaceutical Sciences, Zhejiang Chinese Medical University, Hangzhou, 310053 Zhejiang People’s Republic of China

**Keywords:** RNA nanoparticles, XBP1, siRNA, TNBC, Chemoresistance

## Abstract

**Background:**

To date, triple-negative breast cancer (TNBC) treatment options are limited because of the loss of target receptors and, as a result, are only managed with chemotherapy. What is worse is that TNBC is frequently developing resistance to chemotherapy. By using small interfering RNA (siRNA)-based therapeutics, our recent work demonstrated X-box-binding protein 1 (XBP1) was linked to human epidermal growth factor receptor 2 positive (HER2+) breast cancer development and chemoresistance. Given the instability, off-target effects, net negative charge, and hydrophobicity of siRNA in vivo utilization and clinical transformation, its use in treatment is hampered. Thus, the development of a siRNA-based drug delivery system (DDS) with ultra-stability and specificity is necessary to address the predicament of siRNA delivery.

**Results:**

Here, we assembled RNase resistant RNA nanoparticles (NPs) based on the 3WJ structure from Phi29 DNA packaging motor. To improved targeted therapy and sensitize TNBC to chemotherapy, the RNA NPs were equipped with an epidermal growth factor receptor (EGFR) targeting aptamer and XBP1 siRNA. We found our RNA NPs could deplete XBP1 expression and suppress tumor growth after intravenous administration. Meanwhile, RNA NPs treatment could promote sensitization to chemotherapy and impede angiogenesis in vivo.

**Conclusions:**

The results further demonstrate that our RNA NPs could serve as an effective and promising platform not only for siRNA delivery but also for chemotherapy-resistant TNBC therapy.
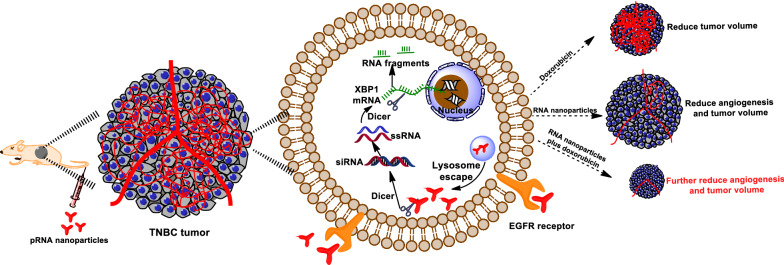

## Introduction

TNBC, which accounts for 12–18% of breast cancer patients, is a more aggressive subtype of breast cancer with poor prognosis and overall survival (OS) [1]. TNBC is negative for estrogen, progesterone, and HER2 receptor and therefore is not eligible for commonly used targeted therapy. Anthracyclines (doxorubicin e.g.,) and taxanes (paclitaxel e.g.,) based neoadjuvant chemotherapy (NAC) is the standard therapy method to manage TNBC [2]. However, due to chromosomal instability, about 50% NAC treated patients evolve chemoresistance [3]. Our recent results show XBP1 is involved in HER2 + breast cancer development and chemoresistance [4]. XBP1 was also reported to regulate a hypoxia inducible factor 1 subunit apha (HIF1α)-dependent transcriptional network in TNBC cells, which facilitates TNBC progression and chemoresistance [5]. Meanwhile, XBP1 expression shows a positive correlation with cell survival of TNBC [6, 7]. However, there is still no related drug, which targeting both TNBC and XBP1 gene to sensitize TNBC to chemotherapy.

siRNA is widely used to understand gene function by targeting specific mRNA expression and has recently been used against various diseases. In November 2019 the second siRNA-based drug has been approved by the FDA, which represents a landmark event for the advancement of RNA-based medicines [8]. However, due to its instability, net negative charge, and hydrophobicity and, in vivo utilization of siRNA remains stagnant [9]. Over the past 20 years, the novel siRNA DDS developed rapidly, including cationic polymer-based [10], exosome-based [11], membrane-camouflaged [12], lipid-based [13], and nanogel-based [14]. Unfortunately, present DDS possess unique drawbacks (such as large particle size, cytotoxicity, aggregation in vivo, and immune response), which impedes in vivo application [15–17]. Recently, our results showed that using a phi29 bacteriophage-derived packaging RNA (pRNA) could deliver the siRNA with high-efficiency [4]. The RNA nanotechnology was developed in 1998 [18] and has been widely used in cancer therapy [19–21]. Compared to other DDS, this pRNA nanoparticles branched ratchet shape, and favorable size (about 10 nm) facilitates tumor penetration [22, 23]. The RNA NPs equip with tumor targeting aptamer can ensure NPs bind to tumor cells with little in healthy organs [24, 25]. At the same time, its electronegativity prevents nonspecific cell targeting, which results in minimum cytotoxicity and off-target effects [26]. Excitingly, chemical modifications of RNA bases can confer RNA NPs more stability to against serum and RNase [27]. At last, RNA NPs were composed of RNA that is easy to synthesize and induces a minimal adverse immune response [28].

In this study, we set out to target therapy TNBC and sensitize TNBC to chemotherapy by using RNA nanotechnology. We found that after intravenous injections of macromolecular pRNA NPs dramatically suppressed breast cancer growth and promoted the sensitization of chemotherapy in TNBC mouse models. XBP1 deletion impaired angiogenesis and combined treatment with RNA NPs and doxorubicin (dox) can further impair tumor angiogenesis and down-regulate HIF1α target expressions. Our findings imply that a macromolecular pRNA platform could efficiently deliver similar macromolecular siRNA. This may offer an effective and promising path for chemotherapy resistant TNBC treatments in the future.

## Experimental section

### Cell culture

Breast cancer cell lines MDA-MB-231 and MDA-MB-453 and normal mammary epithelial cells MCF-10A, 184B5 cells were purchased from Chinese Academy of Sciences Cell Bank (Shanghai, China).

MDA-MB-231, MDA-MB-453 were cultured in Leibovitz’s L-15 medium (Gibco) supplemented with 100 units/mL penicillin streptomycin, 10% FBS (Gibco) and kept at 37 ^o^C in humidified 100% atmosphere. 184B5 cells were cultured in DMEM (Gibco) supplemented with 10% FBS and antibiotics and kept at 37 ^o^C, 5% CO_2_. MCF10A cells were cultured in MEGM (Lonza) supplemented with 100 ng/mL cholera toxin, supplements and growth factors (Lonza) (BPE, hEGF, Insulin, Hydrocortisone, GA-1000), and kept at 37 ℃, 5% CO_2_.

### Generation of 3WJ-EGFRapt-siXBP1 NPs

Multifunctional pRNA-EGFRapt-siXBP1NPs were constructed by a bottom-up self-assembly approach [29]. Briefly, NPs were assembled by mixing the equal molar amounts of RNA fragments and gradually annealing from 95 to 4 ℃ in 1 × RNA annealing buffer (TMS buffer) on a PCR machine.

The therapeutic pRNA-EGFRapt-siXBP1 is composed of four strands. Lowercase letters indicate 2’-F modified nucleotides, and other sequences are adopted as previously described [4].

Strand 1: 5′-uucuuucGAucucuGGcAGuu-3′; Strand 2: 5′-cuGccAGAGAucGAAAGAAuuuuGccAuGuGuAuGuGGG-3′; Strand 3: 5′-ccc AcA uAc uuu Guu GAu ccG ccu uAG uAA.

cGu Gcu uuG AuG ucG Auu cGA cAG GAG Gc-3′ (underlined sequence is EGFR aptamer) [30]; Strand 4: 5′-GGAucAAucAuGGcAA(Cy5)-3′.

The control pRNA-EGFRapt-siScramble is composed of strands with siScramble sequence instead of siXBP1 sequence. Lowercase letters indicate 2’-F modified nucleotides. The siScramble sequence is as follows:

Sense: 5′-GccAGAAAcGGuAcAAGuA-3′; Antisense: 5′-uAcuuGuAccGuuucuGGc-3′.

### Characterization of pRNA-EGFRapt-siXBP1 NPs

The size and zeta potential of our NPs were determined by DLS. To determine the size of NPs, NPs were filtered by 0.22 µm filter and then 100 µL NPs were (20 µM) diluted into TES buffer. To determine the zeta potential, the above NPs were further diluted to 500 µL TES buffer. The T_m_ value was detected by a SYBR green assay, as previously described [4]. Briefly, the NPs were assembled in 1 × TMS buffer in the presence of 1 × SYBR Green II dye (Invitrogen) with final nanoparticle concentration of 250 nM. The samples were heated to 95 °C for 5 min and then slowly cooled down to room temperature at a rate of 0.11 °C/s using the Roche Lightcycler 96 real-time PCR machine. The melting temperature was obtained from at least three independent measurements. As for the stability assay, the 2’F modified NPs or unmodified 3WJ control NPs were exposed to different concentrations of RNase A (0, 10, 100 µg/mL), or 10% FBS supplemented DMEM medium at 37 ^o^C, respectively. The assembly and stability of our NPs was examined through 8% native PAGE gel electrophoresis.

#### In vitro cell binding and uptake

Flow cytometry and confocal microscope were used to evaluate the cellular binding and uptake of NPs. To do this, MDA-MB-231 cells were grown on slides overnight in L15 medium and incubated with 50 nM Cy5-conjugated NPs for 24 hrs at 37 ^o^C. After washing three times with pre-cooling PBS, cells were fixed and stained with cytomembrane dye Alexa488-wheat germ agglutinin (Thermo Fisher) and DAPI (Sigma). The slides were imaging using a Nikon A1 Confocal Microscope System. For flow cytometry analyses, 50 nM Cy5 labeled NPs were incubated with MDA-MB-231 cells for 1, 3, 6, 12 and 24 hr(s). After that, the cells were washed and analyzed by a Cytoflex flow cytometer (Beckman).

#### Specific binding in mixture system in vitro

To evaluate the specific binding of NPs in vitro, Dio pre-staining MDA-MB-231 cells were mixed with the same amount of normal mammary epithelial cells (MCF10A and 184B5) or control tumor cells (MDA-MB-453) with slightly rotating. Following this, 50 nM Cy5-conjugated NPs were added to the mixture and further incubated at 37 ^o^C for 2 hrs. After that, the mixture was collected and analyzed by flow cytometer. The binding efficiency was calculated using the following formula:

Binding efficiency = ratio of Cy5 positive MDA-MB-231 cells/ratio of total MDA-MB-231 cells in mixture × 100%. The NPs binding efficiency was also represented by the value of MFI of Cy5 positive cells.

### Cell viability, cell cycle and apoptosis assays

MDA-MB-231 cells were treated with 50 nM pRNA-EGFRapt-siXBP1 NPs or pRNA-EGFRapt-siScramble control. The cells viability, cell cycle and apoptosis were analyzed after three days culture. Cell viability was measured using the CCK8 assay (Dojindo), following the manufacturers protocol. In a dox sensitivity experiment, MDA-MB-231 cells were treated with dox (2, 0.5 and 0.25 µg/mL, respectively) alone, or with 50 nM NPs for 72 hrs. Then the cell viability was measured using the same method. To analyze cell cycles, cells were incubated with NPs for 72 hrs. After that, cell pellets were collected, permeabilized with 70% (v/v) ethanol, and re-suspended in 1 mL of PBS containing 1 mg/mL RNase and 50 mg/mL propidium iodide (PI). They were then incubated in the dark for 30 min at room temperature and analyzed by Cytoflex Flow Cytometer (Beckman). The cell cycle distribution was evaluated on DNA plots using a MODFIT software. To test apoptosis, annexin V and 7AAD staining (Southern biotech) was performed by flow cytometry.

### Colony formation assay

The soft agar colony formation assay was performed as previously described with slight modifications [31]. 1 × 10^3^ pRNA-EGFRapt-siXBP1 NPs treated breast cancer cells were transferred to 0.8% agarose with the same volume ratio (1:1) in 10% growth medium to make a final concentration of 0.4% agarose. The cell mixture was plated on top of 0.8% bottom layer of agar in the 10% growth medium in 6-well plates. Cells were fed every 5 days for 20 days with 10% growth medium containing 0.4% agarose. For the dox sensitivity experiment, the breast cancer cells were firstly treated with 0.01 µg/mL dox alone, or dox plus prepared NPs for 48 hrs. Once completed the treated breast cancer cells were mixed with 0.4% agarose and seeded to the top of a solidified layer for further incubation. The experiment was repeated in triplicate and the statistical significance was calculated using Student’s t test.

### Real-time PCR

Total RNA was extracted from cultured cells or tumor tissues using a RNeasy mini kit (Qiagen), and reverse-transcription was performed by using HiScript II QRT SuperMix for qPCR (Vazyme Bioteche). Real-time PCR was performed as described previously [4].

### Western blotting

Cells were lysed using RIPA buffer (CST) supplemented with cocktail protease inhibitors (Roche) and PMSF. Minced tumor tissues were homogenized in liquid nitrogen in RIPA buffer. 30 µg of total protein were separated by SDS-PAGE and the separated proteins were transferred to PVDF membranes (Millipore) for western blotting analysis using anti-XBP1 (Abcam, ab37152); anti-β-actin (CST).

### Orthotopic xenograft breast tumor mouse model

Six-week-old female athymic nude mice were purchased from Beijing Vital River Laboratory Animal Technology, and all animal procedures were performed under IACUC-approved protocols at the Zhejiang Chinese Medical University. Athymic nude mice were orthotopically injected with 1 × 10^7^ MDA-MB-231 cells mixed with Matrigel into the fat pads of the fourth pair of mammary glands. When the tumor sizes reached 100–150 mm^3^, the mice were randomly divided into five groups (six/group) and intravenously injected with pRNAs (3.3 nmol/mice) twice a week for 3 weeks. For dox treatments, dox (Sigma) (3 mg/kg) was intravenously injected into mice twice a week for 3 weeks and 1 day before pRNA injection. When the tumor sizes reached 1000 mm^3^ (that is 25 days post-injection in this study), mice were sacrificed, and the tumors were collected, then lysed for extraction of total proteins and RNAs, or fixed in 10% neutral buffered formalin, respectively, for further Western blot, real-time PCR and IHC studies, etc. The tumor volume was measured, and tumor size was calculated using the formula: volume = 0.5 × (width)^2^ × (length).

### Histochemical analysis

To examine the biodistribution of NPs within the tumor issues, O.C.T.-embedded frozen sections (5 µm) were examined by confocal microscopy. To analyze tumor cell blood vessels, tumor tissue samples were collected and fixed in 10% neutral buffered formalin, followed by dehydration via a gradient series of ethanol (75%, 85%, 95% and 100%) and embedded in paraffin following routine methods. For CD31 immunostaining, slides were blocked with 3% H_2_O_2_-methanol for 15 min at room temperature, treated with mouse anti-CD31 antibody (Abcam, ab28364) overnight at 4 °C, with horseradish peroxidase (HRP)-conjugated secondary antibody, and with 3, 3′-diaminobenzidine (DAB). The tissue sections were counterstained with hematoxylin for nucleus visualization. The slides were analyzed on an LSM 510 Meta confocal microscope.

## Results and discussion

### Synthesis and characterization of pRNA-EGFRapt-siXBP1 NPs

**Fig. 1:**
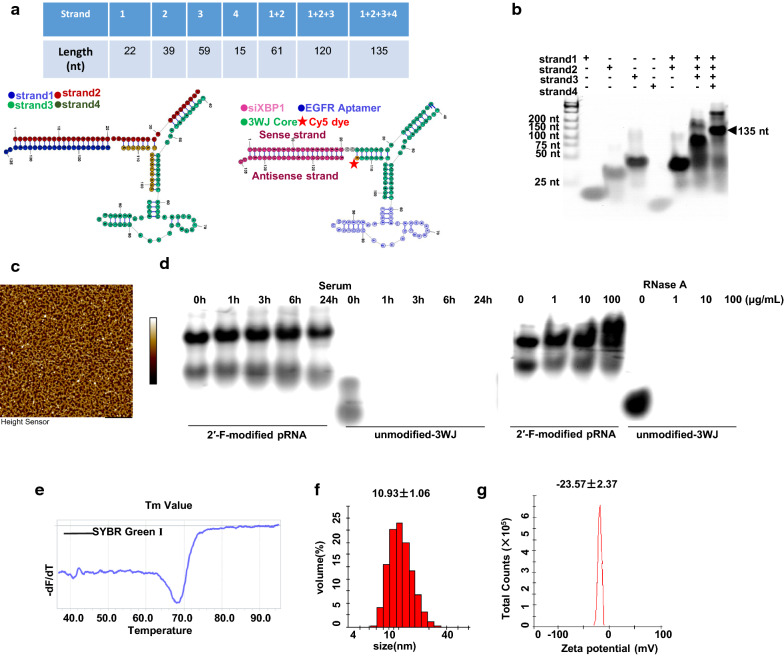
Characterizations of pRNA-EGFRapt-siXBP1 NPs. **a** Scheme of pRNA-EGFRapt-siXBP1NPs. **b** Native PAGE showing stepwise highly efficient assembly of the NPs. **c** Atomic force microscopy (AFM) images of pRNA nanoparticles. **d** Stability analyze by 8% native PAGE gel electrophoresis after RNase A and10% FBS-supplemented DMEM medium treatments for the indicated time at 37 °C. **e** Tm value of pRNA-EGFRapt-siXBP1 NPs determined by SYBR Green assay. **f** DLS measurements showing the hydrodynamic size. **g** Zeta potential

Throughout the remainder of the text, these NPs are denoted as pRNA-EGFRapt-siXBP1. Our NPs harboring 2’-F modified EGFR RNA aptamer and therapeutic siXBP1, using Cy5 fluorophore as marker, were composed of four short fragments (Fig. 1a). The RNA oligonucleotides were synthesized chemically (Genscript) then mixed in an equal molar ratio in TMS buffer and annealed to generate uniform NPs. 8% Native PAGE gel assays show the highly effective stepwise assembly of the complex (Fig. 1b), which is in line with the AFM result (Fig. 1c). The stability assay of 2’-F-modified NPs were evaluated in 10% FBS and different concentrations of RNase by naive PAGE gel assay. Compare to unmodified NPs, 2’-F-modified NPs can be resistant to 100 µg/mL RNase and were stable in 10% FBS (Fig. 1d). Alongside chemical modification, other NPs packaging (such as iron oxide magnetic-based NPs) can also impart higher resistance to serum and RNase to increase RNA stability [32, 33]. NPs with high thermodynamic stability prevent dissociation at ultra-low concentrations in vivo. The Tm value of our NPs is 68.2 ^o^C (Fig. 1e) determined by the LightCycler® 96 Real-Time PCR System (Roche), which means a higher stability of RNA duplex [34]. The average hydrodynamic diameter of NPs was 10.93 nm (Fig. 1f). This size is larger than renal depletion cutoff sizes, while small enough to minimize macrophage phagocytosis. This size allows NPs penetrate into tumor tissues via the EPR effect and receptor-mediated endocytosis when targeting ligands are equipped [35]. RNA NPs are indeed highly negatively charged, and this is reflected in the zeta potential measurements showing a single peak at -23.57 mV for NPs (Fig. 1g).

### pRNA-EGFRapt-siXBP1 NPs efficiently and specifically target and bind to TNBC cells in vitro

**Fig. 2:**
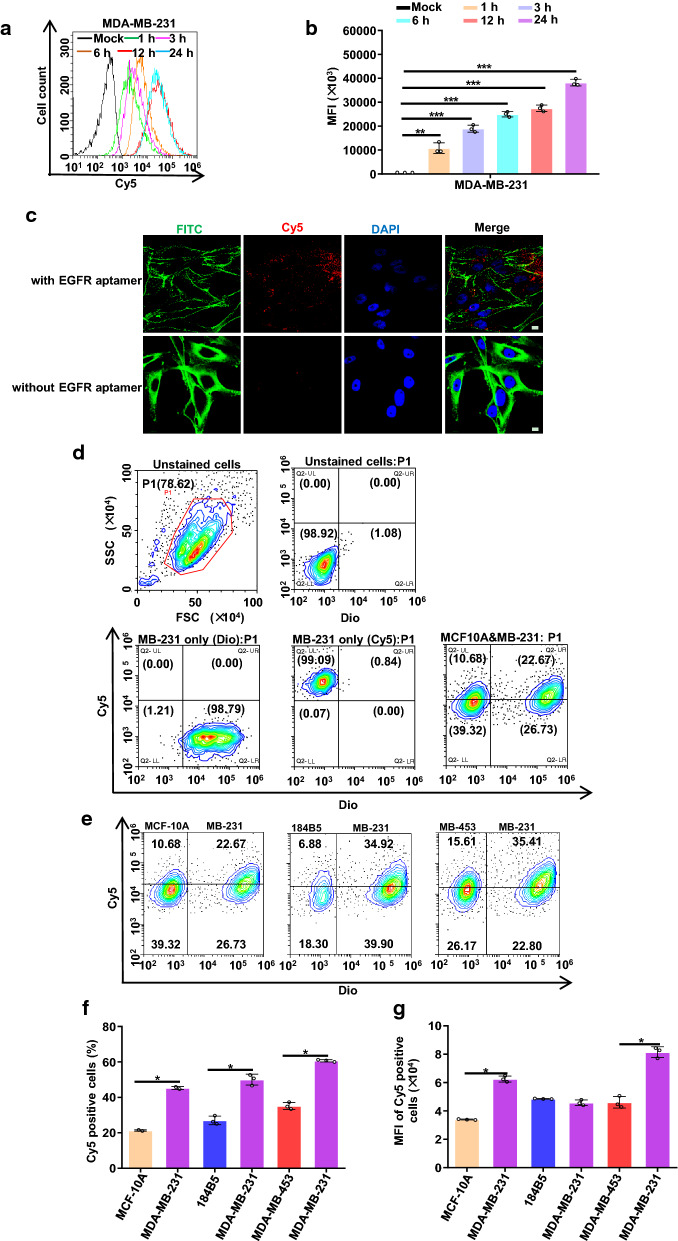
pRNA-EGFRapt-siXBP1 NPs cell uptake, specific binding to tumor cells in vitro. **a**, **b** Cell binding is assessed by flow cytometry. **c** Confocal images showing efficient binding and internalization into MDA-MB-231 cells. Green: cell membrane; blue: nuclei; and red: RNA nanoparticles. Scale bars, 10 µm. **d** Representative gating strategies in mixture of MDA-MB-231 and MCF10A cells. **e** Cell specific binding is assessed by flow cytometry. **f** pRNA-EGFRapt-siXBP1 NPs binding efficiency were evaluated. **g** MFI of Cy5 positive cells

Compare with other subtypes of the breast cancer, EGFR is frequently overexpressed in TNBC, thus making it a potential target for TNBC targeted therapy [36]. Aptamers, often termed ‘chemical antibodies’, are functionally equal to protein antibodies, but with more advantages, such as ease of modification, chemo-synthesis, high stability and extremely mininal immunogenicity. While they are capable of blocking cell surface receptors they can also deliver therapeutic agents into cells [37]. Here, we assemble NPs with EGFR aptamer, and evaluated NPs binding efficiency by flow cytometry. MDA-MB-231 is a highly aggressive, invasive and poorly differentiated TNBC cell line as it lacks ER and PR expression, as well as HER2 amplification. Similar to other invasive cancer cell lines, the invasiveness of the MDA-MB-231 cells is mediated by proteolytic degradation of the extracellular matrix [1]. As one of the most popular TNBC cell line, MDA-MB-231 cell line was proved to be resistant to chemotherapy [38]. MDA-MB-453 cell line was EGFR negative cell line and was classed as a ‘HER2 over-expression’ breast cancer cell line. 184B5 is normal human mammary epithelial cells and express high level of insulin receptor but not EGFR [39]. The overlay histogram (Fig. 2a) and median fluorescence intensity (MFI) (Fig. 2b) results indicate that pRNA-EGFRapt-siXBP1 NPs efficiently bind to MDA-MB-231 cells. Confocal microscopy images further confirm the efficient binding and internalization of pRNA-EGFRapt-siXBP1 NPs into target cells after 24 hr incubation (Fig. 2c).

To further confirm the binding specificity in vitro and mimic NPs binding to tumor cells in the presence of normal mammary epithelial cell in vivo, Dio pre-staining MDA-MB-231 cells were mix with MCF10A,184B5, and MDA-MB-453 cells, respectively. Flow cytometry was used to evaluate the specific binding ability of pRNA-EGFRapt-siXBP1 NPs to MDA-MB-231 cells in the mixture system (Fig. 2d and e). The ratio of Cy5 positive MDA-MB-231 cells to total MDA-MB-231 cells and MFI in MAD-MB-231 cells was significantly higher than that of non-target cells (Fig. 2f g) (*p* < 0.05). These results indicate pRNA-EGFRapt-siXBP1 NPs specific binding ability in vitro and imply its specific binding ability in vivo.

### XBP1 gene knockdown sensitizes MDA-MB-231 cells to doxorubicin treatment in vitro, reduces cell viability, impairs mammospheres forming ability, but does not alters cell apoptosis and cell cycle

**Fig. 3:**
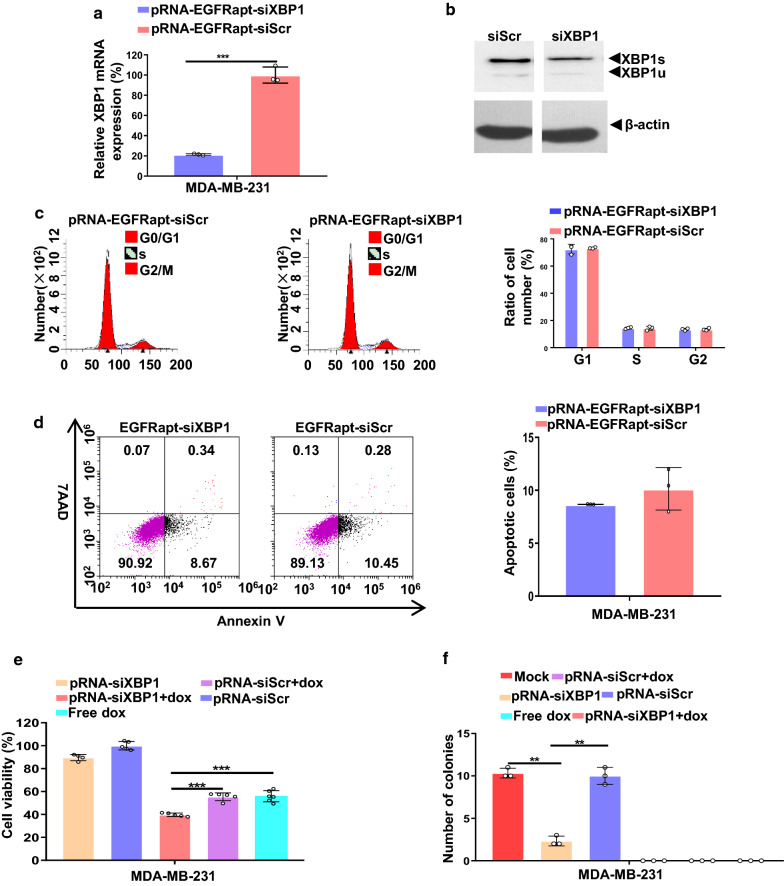
pRNA-EGFRapt-siXBP1 NPs silenced XBP1 expression, sensitizes MDA-MB-231 cells to doxorubicin treatment in vitro, reduces cell viability, impairs mammospheres forming ability, but not alters cell apoptosis and cell cycle. **a** RT-PCR result of XBP1 gene expression in MDA-MB-231 cells after treated with 50 nM NPs. (mean ± s.d., n = 3). ***, *p* < 0.001. **b** Western blot analysis of XBP1s and XBP1u expression in MDA-MB-231cells after pRNA-EGFRapt-siXBP1 NPs treatment for 72 h. **c** Flow cytometry analysis of MDA-MB-231 cells cell cycle alteration after treated by pRNA-EGFRapt-siXBP1 or pRNA-EGFRapt-siScr control, respectively. (mean ± s.d., n = 3). **d** Cell apoptosis determined by annexin V and 7AAD staining. **e** Cell viability analysis of MDA-MB-231 cells after treated by NPs or NPs plus dox for 72 h. (mean ± s.d., n = 5–7). ***, *p* < 0.001. **f** Quantification of soft agar colony formation in different groups. (mean ± s.d., n = 3). **, *p* < 0.01. **

Our cell-binding results imply NPs could efficiently silence XBP1 expression. To verify this speculation, we firstly evaluated XBP1 mRNA and protein expression in NPs treated MDA-MB-231 cells. XBP1 mRNA expression is reduced by 80% after 50 nM pRNA-EGFRapt-siXBP1 treatment for 72 hr (Fig. 3a). In line with the XBP1 mRNA level decrease, XBP1 proteins expressions were also decreased (Fig. 3b). It should be known that in TNBC, the effect of XBP1 silencing on cell cycle is still no studied. Our previous data showed that XBP1 knock-down in HER2 + breast cancer cell lines results in cell cycle arrest in S phase [4]. Here, we firstly show that XBP1 silencing in TNBC cannot induce cell cycle alternation (Fig. 3c). Cell cycle alternation trigger cell death pathways [40]. We further measured cell apoptosis changes after XBP1 silencing by an Annexin V and 7AAD assay (Fig. 3d). Different with HER2 + breast cancer, the result suggests XBP1 silencing do not alter cell apoptosis (Fig. 3d) [4]. As XBP1 knockdown has recently been reported to sensitize TNBC to doxorubicin (dox) treatment by siRNA interference [5], we next examined if our NPs treatment could sensitize TNBC to dox treatment in vitro. We found that treatment with NPs alone could slightly reduce cell viability declines in MDA-MB-231 cells (Fig. 3e). pRNA-EGFRapt-siXBP1 NPs and dox combined treatment could significantly reduce cell viability than dox treatment alone (Fig. 3e). We next tested the effect of pRNA-EGFRapt-siXBP1 NPs on soft agar colony forming of MDA-MB-231 cells. Compared to pRNA-EGFRapt-siScramble control and mock group, pRNA-EGFRapt-siXBP1 NPs pretreatment highly impaired soft agar colony forming ability of HER2 + breast cancer cells (*p* < 0.01) (Fig. 3f). Notably, dox treatment can completely abolish MDA-MB-231 cells colony forming capacity (Fig. 3f). These results indicated that combined utilization of pRNA-EGFRapt-siXBP1 NPs could sensitize the TNBC to dox treatment in vitro.

### pRNA-EGFRapt-siXBP1 NPs specifically bind to tumor cells and efficiently silence XBP1 expression in vivo

**Fig. 4:**
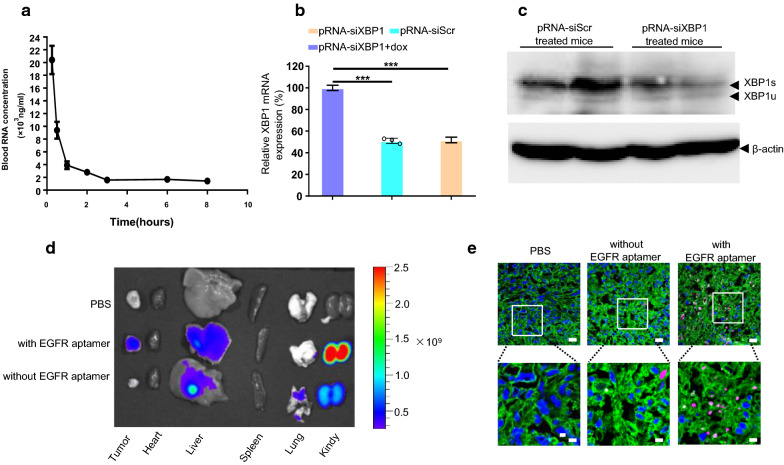
pRNA-EGFRapt-siXBP1 NPs specifically bind to tumor cells and efficiently silence XBP1 expression in vivo. **a** Pharmacokinetic study of pRNA-EGFRapt-siXBP1. **b** Quantitative RT-PCR analysis of XBP1 expression in MDA-MB-231 xenograft tumor. Data are presented relative to β-actin. n = 3. **c** Western blot analysis of XBP1s and XBP1u expression in MDA-MB-231 tumor mouse. **d** Biodistribution of Cy5-labeled pRNA-EGFRapt-siXBP1 NPs 16 h post intravenously injection. **e** Confocal microscopic images of tumor sections from mice injected intravenously with pRNA-EGFRapt-siXBP1 or control NPs without EGFR aptamer. Red, Cy5-labelled pRNA; green, Alexa488-wheat germ agglutinin-labelled cell membrane; blue, nuclear staining with DAPI. Scale bars, 20 µm and 7 µm, respectively

Recent cancer treatments have been developed, many are often nonspecific and have toxic side effects on non-targeted tissues. To overcome these challenges, a drug needs an ideal pharmacokinetic (PK) profile to reach targeted cells specifically with little or no accumulation in healthy organs(e.g. off target organs) [41]. The plasma concentration-time plot of pRNA NPs shows a typical two-phase kinetics with an initial rapid distribution phase and a highly prolonged half-life (Fig. 4a). This is consistent with Abdelmawl’s study [42]. To analyze gene silencing capacity in vivo, MDAMB-231 cells were grown subcutaneously in BALB/c immuno-deficient mice. Compared to siScramble treated mice, pRNA-EGFRapt-siXBP1 NPs treatment significantly down-regulated XBP1 expression in both mRNA (Fig. 4b) and protein (Fig. 4c) level in vivo. To confirm pRNA specific binding to tumor in vivo, we constructed a pRNA without EGFR aptamer and test its binding in vivo. To do this, pRNA-EGFRapt-siXBP1 NPs or control pRNA NPs were injected via the tail vein. The tissue distribution image demonstrated that Cy5-conjugated pRNA-EGFRapt-siXBP1 NPs but negative control pRNA NPs without EGFR aptamer were highly accumulated in the area of the xenograft tumor after systemic administration (Fig. 4d). Notably, a strong Cy5 signal was observed in the kidneys. A recent study suggested RNA nanoparticles larger than the upper limit of renal excretion nanoparticles could pass the renal filtration and resume their original structure identified in urine. RNA nanoparticles rubber-like deformation property enables them to squeeze out of the leaky vasculature to improve the EPR effect. RNA nanoparticles rubber-like property also confers them nontoxic to body since they can be rapidly cleared from the body via renal excretion into urine with little accumulation in the body [43]. Importantly, confocal microscopic analyses of frozen tumor sections indicated that pRNA-EGFRapt-siXBP1 NPs effectively penetrated the tumor cells but not control pRNA NPs (Fig. 4e). Various positively charged macromolecules were proven to be effective in siRNA transfection. However, these poly-cations are not suitable for systemic targeted (non-liver) delivery in vivo; this has been a long-standing problem [44]. Here, we demonstrated pRNA NPs without poly-cations materials can specifically bind to tumor cells and efficiently silence XBP1 expression in vivo, which shows huge potential for in vivo utilization of siRNA.

### Inhibition of TNBC growth, impaired angiogenesis, and sensitization to dox by combinational treatment with pRNA-EGFRapt-siXBP1 NPs in vivo

**Fig. 5:**
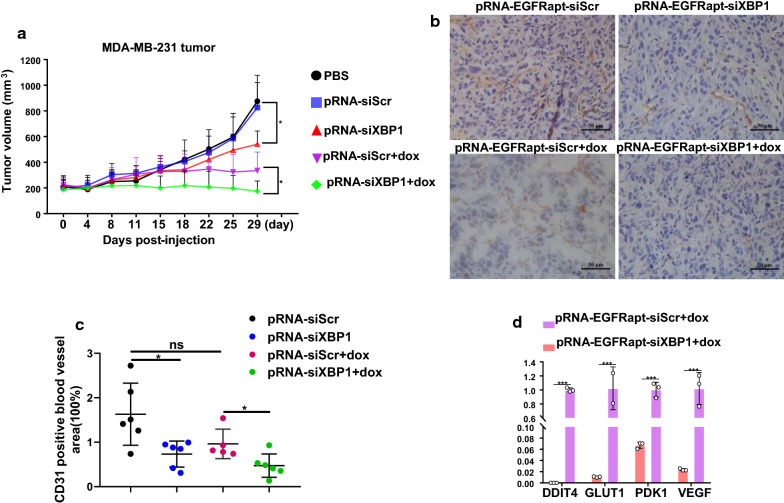
pRNA-EGFRapt-siXBP1 NPs treated inhibits TNBC in vivo. **a** Tumor growth in different group treated TNBC mice. (mean ± s.d., n = 5). *, *p* < 0.05. **b**, **c** CD31immunostaining of TNBC tumor. Scale bars, 50 µm. **d** Quantitative RT-PCR analysis of VEGFA, PDK1, GLUT1, and DDIT4 expression in TNBC tumor after pRNA-EGFRapt-siScr plus dox and pRNA-EGFRapt-siXBP1 plus dox treatment. Results are presented relative to β-actin expression. (mean ± s.d., n = 5). ***, *p < 0.001*

To analyze if XBP1 gene silencing *in vivo e*ffect TNBC growth and chemoresistance, MDA-MB-231-beaing mice were treated with pRNA-EGFRapt-siXBP1 NPs or pRNA-EGFRapt-siXBP1 NPs plus dox. Excitingly, tumor growth of TNBC tumors was significantly inhibited by pRNA-EGFRapt-siXBP1 NPs (*p < 0.05*) (Fig. 5a). Strikingly, the tumor volume was further reduced significantly after pRNA-EGFRapt-siXBP1 plus dox treatment as compared to dox treatment alone group (*p* < 0.05) (Fig. 5a). It was reported that XBP1 can function via growth factor signaling pathways to regulate endothelial proliferation and angiogenesis [45]. Here, we investigated the relationship between XBP1 expression and angiogenesis in TNBC mouse model. Our tumor frozen sections were stained with CD31 antibody and blood vessels area was counted by ImageJ software (Fig. 5b). We confirmed that XBP1 depletion by pRNA-EGFRapt-siXBP1 NPs in TNBC impaired angiogenesis (Fig. 5c) (*p* < 0.05). Although decreased angiogenesis was observed in dox only treated mice, no significant difference was observed. Notably, the blood vessels area was further reduced after pRNA-EGFRapt-siXBP1 NPs plus dox treatment than pRNA-EGFRapt-siScr NPs plus dox group (Fig. 5c) (*p* < 0.05). It should be noted that pRNA-EGFRapt-siScr NPs plus dox group treatment also decreased the blood vessels area, although no significant difference was observed between pRNA-EGFRapt-siScr NPs and pRNA-EGFRapt-siScr NPs plus dox group. Actually, studies suggested DNA-damaging drugs dox could affect VEGF expression and HIF-1 activity in human ovarian cancer and other cancers [46, 47]. It was reported that XBP1 promotes TNBC by regulating the HIF1α pathway [5]. When studying the HIF1α target expressions of pRNA-EGFRapt-siXBP1 plus dox treated MDA-MB-231 tumor, we observed that combined treatment highly down-regulated HIF1α targets *VEGFA, PDK1, DDIT4* and GLUT1 expression than dox alone treated mice (Fig. 5d) (p < 0.001).

## Conclusions

In conclusion, we assembled RNase resistant macromolecular RNA NPs equipped with EGFR targeting aptamer and therapeutic macromolecular siRNA drug to sensitize TNBC to chemotherapy utilizing bacteriophage phi29 derived pRNA. Intravenous injections of RNA NPs with EGFR aptamer result in strongly bounding to tumors and XBP1 silencing in vivo. XBP1 deletion by RNA NPs impaired angiogenesis and dramatically suppressed breast cancer. Importantly, RNA NPs treatment sensitizes TNBC to chemotherapy. We believe that the macromolecular RNA NPs serves as an effective and promising platform for similarly macromolecular siRNA therapeutics delivery. The combination of macromolecular RNA NPs and siRNA can likely be utilized to open new possibilities for targeted and specific treatment of breast cancer and other malignancies.

pRNA-EGFRapt-siXBP1 nanoparticles treatment reduces TNBC volume and angiogenesis. RNA nanoparticle treatment sensitizes TNBC to chemotherapy, which evidence by further decrease of tumor volume and angiogenesis after combined treatment with doxorubicin.
